# Occupational Exposures in the Culinary Underbelly: Air Pollution in Restaurants

**DOI:** 10.1021/acs.est.5c18025

**Published:** 2026-06-21

**Authors:** Antonio F. Saporito, Mehmood Hashmi, Wuyue Yu, Drew R. Michanowicz, Eric D. Lebel, Nicole Lucha, Gan Huang, Colin J. Finnegan, Vittorio Albergamo, Wenjie Lyu, Steven N. Chillrud, James M. Ross, Dru A. Burns, Ryan F. LeBouf, Christopher Galarza, Judith T. Zelikoff, Terry Gordon

**Affiliations:** † Division of Environmental Medicine, 12296 NYU Grossman School of Medicine, New York, New York 10010, United States; ‡ 725446 PSE Healthy Energy, Oakland, California 94612, United States; § Department of Earth System Science, 6429 Stanford University, Palo Alto, California 94305, United States; ∥ Department of Pediatrics, NYU Grossman School of Medicine, New York, New York 10016, United States; ⊥ Lamont-Doherty Earth Observatory, 57699 Columbia University, Palisades, New York 10964, United States; # Respiratory Health Division, National Institute for Occupational Safety and Health, Morgantown, West Virginia 26508, United States; ¶ Forward Dining Solutions LLC|EcoChef, Pittsburgh, Pennsylvania 15147, United States

**Keywords:** occupational health, exposure assessment, restaurant, particulate matter, VOCs, PAHs, mixtures

## Abstract

Occupational exposure to commercial cooking emissions has not been comprehensively studied, particularly in a Western context. This investigation measured the concentration and composition of volatile organic compounds (VOCs), polycyclic aromatic hydrocarbons (PAHs), particulate matter (PM), carbon monoxide (CO), and black carbon (BC) in 18 New York City restaurants. Eight-hour gravimetric PM_1,2.5,10_ mass concentrations were measured in kitchen and dining areas, and PM_1_ was speciated using X-ray fluorescence. Evacuated canisters and XAD-2 sorbent tubes collected VOCs and PAHs, respectively, and were speciated using gas chromatography/mass spectrometry (GC/MS). Lastly, spatial concentration gradients for PM_2.5_ and day-of-the-week effects were considered in eight additional restaurants. A median PM_2.5_ concentration of 79.6 μg/m^3^ was found in kitchens, with real-time values spiking into the thousands of micrograms per cubic meter in some restaurants—values below the Occupational Safety and Health Administration 8 h permissible exposure limit of 5 mg/m^3^ for particles not otherwise regulated (respirable fraction), but well above the 24 h World Health Organization recommended ambient exposure limit of 15 μg/m^3^ for 2021. Benzene, a known carcinogen, was frequently detected in the kitchen areas, while PAHs were often undetected. These data warrant further investigation into the cardiopulmonary health of restaurant workers.

## Graphical abstract

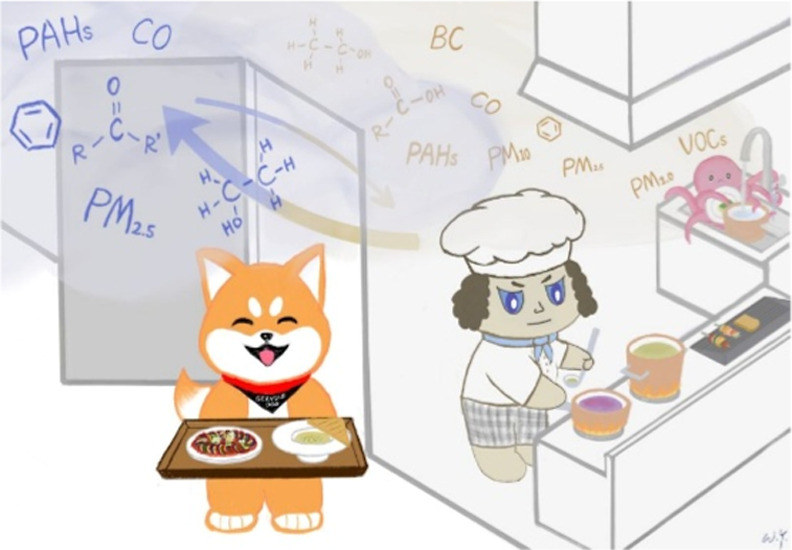

## Introduction

Air pollution often results from fuel combustion or industrial processes and is concentrated in places of high anthropogenic activity. The commercial cooking environment is no exception. Laboratory experiments have indicated that complex mixtures of pollutants exist in kitchens whereby high temperatures and cooking techniques can volatilize or aerosolize potentially toxic compounds.^1^ Dubbed “cooking emissions”, “cooking effluent”, or “cooking oil fumes”, these unhealthy, breathable pollutants are often composed of particulate matter (PM), polycyclic aromatic hydrocarbons (PAHs), volatile organic compounds (VOCs), black carbon (BC), nitrogen compounds (NO_x_), trace metals, and other organic compounds (e.g., heterocyclic amines, aldehydes, ketones).^2–4^

Heating processes in cooking environments, including grilling, frying, roasting, sautéing, searing, baking, and toasting—both a tasty and nonexhaustive list—have been shown to generate PM. In short, thermal degradation of oils, proteins, and carbohydrates produce smoke containing mutagenic organic compounds. High temperature fuel combustion generates NO_*x*_ molecules that can react with VOCs to form secondary organic aerosols (SOAs). Inefficient combustion of fuel or degradation of cooking oils can produce carbonaceous particles. All of these products are primarily generated in the ultrafine region, but rapidly agglomerate to larger sizes depending on humidity, temperature, and air movement.^1–4^

PAHs are a class of organic molecules that can partition from the gas phase into PM by condensation or by adsorbing onto existing particles. They may also be directly emitted as primary organic aerosols. This broad class of mutagenic aromatic hydrocarbons is a product of incomplete combustion or molecular decomposition of organic material at high temperatures. PAHs have a range of volatility, but many species are considered semivolatile (SVOCs), with lower volatility compounds partitioning to the gas phase only under high temperatures and low air pressure. Cooking fatty meats, grilling, biomass burning, and the use of charcoal tend to generate the largest amounts of PAHs and aromatic compounds compared to other techniques and food combinations.^4–6^

Deep frying, sautéing, and other cooking methods involving oil decomposition also release vaporous PAHs and SVOCs but largely generate intermediate volatiles (IVOCs) and VOCs. IVOCs have slightly higher vapor pressure than SVOCs and often vaporize and react in the gas phase. These reactants are precursors for SOAs, and the products often condense into particles. In contrast, VOCs are a class of gaseous compounds with the highest vapor pressures. VOCs released into the kitchen air from decomposing fats and oils can include small mutagenic compounds such as aldehydes (e.g., acetaldehyde, acrolein) and ketones (e.g., acetone, 2-butanone).^7–10^ Additionally, natural gas burners have been shown to leak VOCs such as hexane and toluene, with benzene postcombustion, whereas industrial cleaners can often off-gas ethanol, isopropanol, d-limonene, and methylene chloride into the environment.^11–13^

The use of gas appliances and various culinary techniques may thus produce a diverse profile of pollutants, and these can be modified by cuisine type, oil composition, temperature, and other factors. Most literature has focused on Eastern-style cooking, where high temperature propane-burning and wok stir-frying methods can emit a different profile of aerosols than from those of their Western counterparts, defined by grilling and roasting.^14–16^ Eastern-style has been shown to emit higher concentrations of PM_2.5_ than Western i.e., 30–1400 vs 20–535 μg/m^3^, respectively. Meanwhile, in a study by Zhao et al. 2007b, Western style cuisine produced more organic components, while PAHs were emitted in higher concentrations in Eastern cooking (2855 vs 40 ng/mg, respectively).^17^ Studies by Li et al. 2019,^15^ and Zhao et al. 2007c have found similar patterns.^16^

High-volume exhaust hoods, specialized baffles, and deep fryer flues are critical in removing the majority of kitchen produced gases and aerosols. However, the efficiency of these systems to scrub cooking emissions is highly dependent on proper usage, regular cleaning, and compliance with regulations. Research regarding the breathable components of the commercial kitchen environment have indicated cooking staff are exposed despite these mitigation strategies. Lai et al. found exposure to cooking oil fumes significantly increases the urinary concentration of PAH metabolites in military cooks.^18^ Similarly, Pan et al. also found that increased exposures to PM and PAHs were correlated to oxidative damage in culinary workers.^19,20^ In 2002, Svendsen et al. characterized aerosolized fat aerosols and aldehydes in Norwegian kitchens, and subsequent 2009 and 2013 investigations indicated substantial personal exposure to PAHs and ultrafine particles.^21–23^ Recent international studies including those from Korea,^24^ India,^25^ Iran,^26^ Thailand,^27^ and Turkey^28^ have also indicated restaurant-generated PM, VOCs, and PAHs exposure from cooking, but importantly, these cuisines are often accompanied by regionally specific cooking techniques. Significantly less research has been conducted on aerosols in Western-style commercial kitchens, which particularly in the U.S., remain to be comprehensively characterized.

Restaurant professionals work in dynamic, high-output environments where the industry standard for a chef’s workweek is notoriously long, often surpassing 40 to 60 h.^29–31^ These chronic exposures put back-of-house (kitchen) and possibly front-of-house (dining) staff at disproportionate risk for adverse health outcomes, notwithstanding potential comorbidities. Such exposure periods, and lack of literature examining these cooking environments, necessitate investigation into these occupational settings. In the U.S., occupational exposure to cooking emissions has yet to be characterized. Therefore, the commercial kitchen may impose unknown health risks on the approximately 12.5 million workers in the food services sector.^32,33^ This study sought to characterize pollutants within Western-style commercial kitchens to better understand the potential for these exposures to influence adverse health outcomes.

## Methods and Materials

### Inclusion and Exclusion Criteria

Restaurants were selected for air quality sampling within the New York City (NYC) Metropolitan area and identified using a Google Maps^34^ search with the keywords “American”, “Diner”, “European”, “Latin”, or “New American” indicating the use of Western-style cooking techniques. Restaurants were excluded if the search coproduced the terms “Tavern” or “Bar”, suggesting cuisine is not the primary aim of the establishment. Restaurants were also excluded if kitchens were operating less than 8 h per day. An 8 h sampling period was chosen to represent a typical full work shift and to capture emissions across at least two meal services. If the restaurant had received substantial violations from the NYC Department of Health and Mental Hygiene^35^ within the past 3 years (below “A” grade), they were excluded. As of 2025, 89.2% of restaurants have received an “A” health grade, suggesting that high food safety standards characterize most NYC restaurants. Restaurant menus were inspected and indicated at least 4 out of the 5 characteristic Western techniques of “grilling, broiling, roasting, deep frying, and stewing” were utilized for inclusion. Furthermore, these restaurants were classified as “full-service” and contained a separate dining area used for comparison. All restaurants had a front door opening to street-level. Supporting Information regarding characteristics of these restaurants is presented in Table S1.

### Exposure Assessment

Sampling at 18 restaurants occurred for an 8 h period in the kitchen with concurrent sampling in the dining area as a reference for comparison. Devices used to measure PM, PAHs, VOCs, CO, and BC were enclosed in a water-resistant Pelican case (Figure 1). In the kitchen, these battery-operated exposure assessment devices were set on a flat surface, at a height of 1.5–2.0 m above the floor level and within 1.5 m horizontally of the cooking stove—typically the centrally located “staging” area—to safely mimic the workers’ exposure environment. The devices in the dining area were located ≥3.0 m away from both the entrance door and kitchen area.

**Figure 1:**
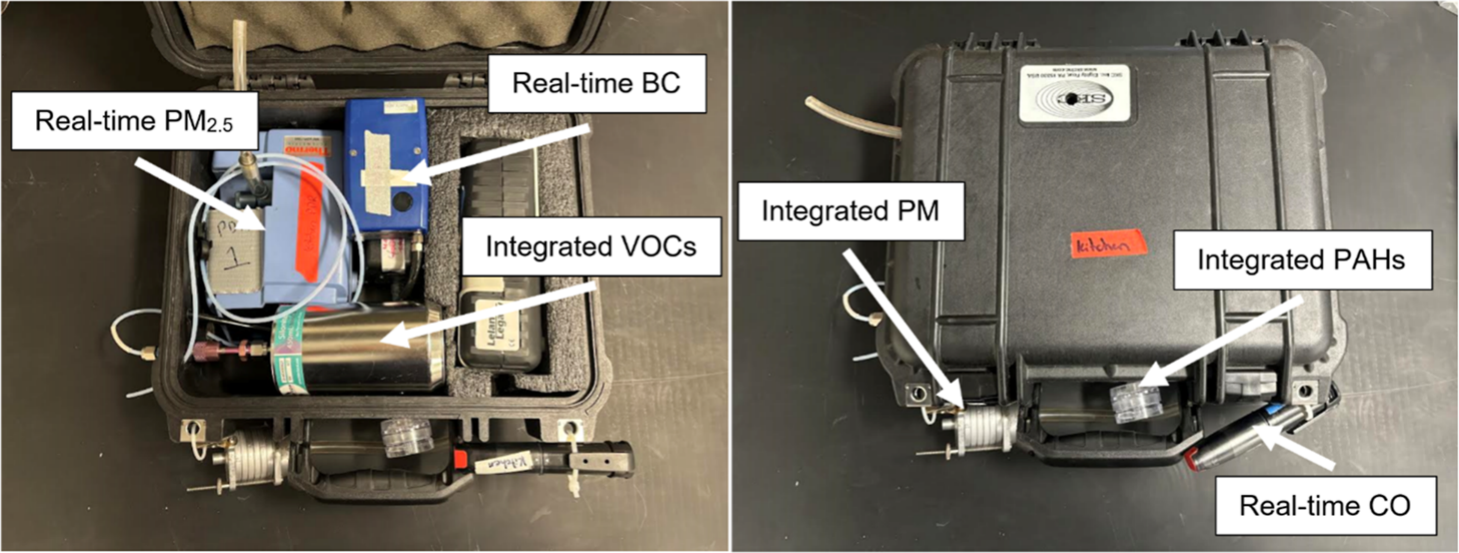
Battery operated sampling instrumentation enclosed in a Pelican case.

### Particulate Matter

PM was collected using a Sioutas cascade impactor with a Leland Legacy pump (SKC) operating at 9 L/min. Fractions of aerodynamic diameters 1–2.5 μm and 2.5–10 μm were collected on 25 mm diameter, 0.5 μm pore size PTFE filters (SKC), whereas PM_1_ was collected on 37 mm diameter, 2.0 μm pore size PTFE filters (Environmental Express). Filters were conditioned in a temperature and humidity controlled chamber (Measurement Technology Laboratories) for ≥24 h prior to and after sampling, and pre/postweighed using an XS105 Mettler scale (Mettler-Toledo Inc.) following the U.S. Environmental Protection Agency’s (EPA’s) *Quality Assurance Guidance Document* 2.12.^36^ All SKC pumps were calibrated using a DryCal Defender 530+ (Mesa Laboratories, Inc.) prior to each sampling period. For comparison, outdoor ambient PM_2.5_ concentrations were averaged during the corresponding 8 h sampling period from the 12 local New York State’s Department of Environmental Protection monitors within NYC. Data were accessed on February 14th, 2025.

**Figure 2:**
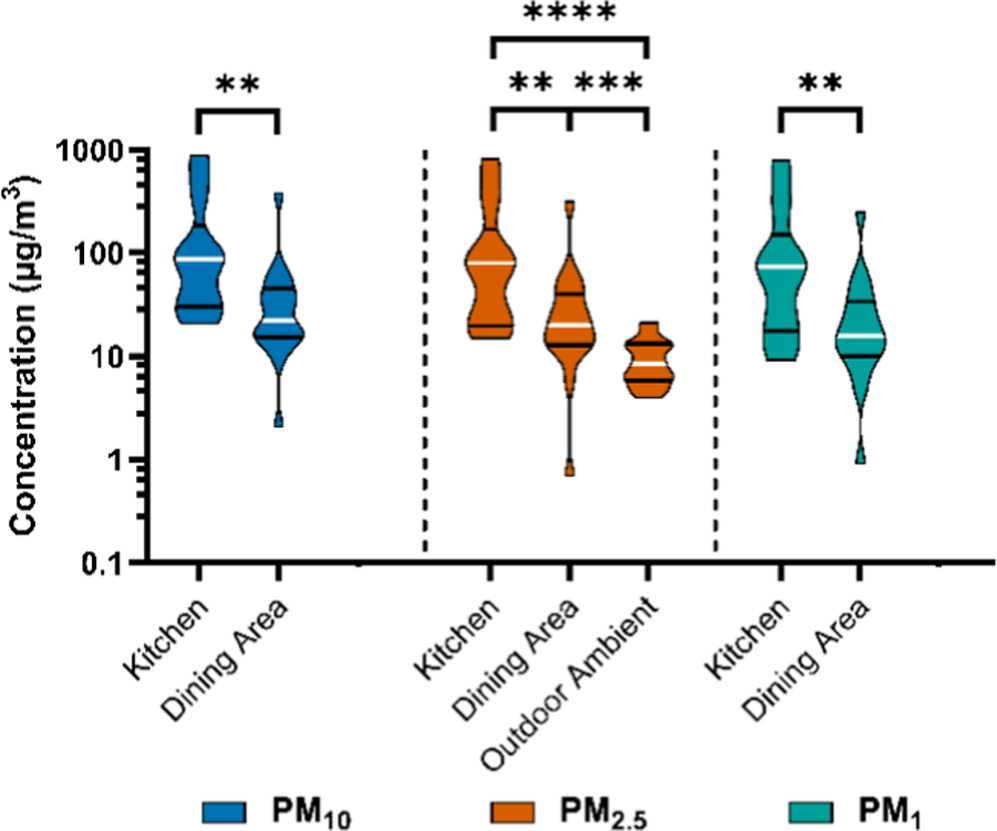
Violin plots of gravimetric PM concentrations (log scale) of different aerodynamic diameters for *n* = 17 kitchens, *n* = 18 dining rooms, along with ambient PM_2.5_ averages *n* = 18. Median denoted by a white line and quartiles by a black line. Statistical significance determined via Mann–Whitney *U* test. ** for *P* ≤ 0.01, *** for *P* ≤ 0.001, and **** for *P* ≤ 0.0001.

PM_1_ was speciated for trace element concentrations on the PTFE filters via ARL QUANT’X energy dispersive X-ray fluorescence (EDXRF; Thermo Scientific) at the Lamont-Doherty Earth Observatory of Columbia University, Palisades, NY, USA, calibrated with four different loadings of metal mixtures on PTFE filters. Briefly, data for each measurement that were <3× the reported EDXRF uncertainty values were set to zero. For data greater than machine uncertainty, field blank concentrations were subtracted from the sample concentrations. Values below the limit of detection (LOD, 1.65σ) were substituted with LOD/2 for descriptive statistics.

Real-time PM_2.5_ concentrations (see Supporting Information) were measured at 1 min intervals using a Personal DataRAM pDR-1500 Aerosol Monitor (Thermo Scientific). Prior to sampling, aerosol monitors were zeroed with a high-efficiency particulate air (HEPA) filter. Post sampling, real-time mass concentrations were calibrated to the colocated gravimetric PM_2.5_ samples and the correction factor utilized for conversion of the light scattering aerosol pDR-1500 measurements. Additionally, EL-USB-CO (Lascar Electronics) was used to collect minute-by-minute concentration data for CO.

A microAeth AE51 (AethLabs) operating at 150 mL/min was used to collect minute-by-minute concentration data for BC. For comparison, 37 mm PM_1_ filter black smoke (BS) concentrations were measured using a model 43D smoke stain reflectometer (SSR; Diffusion Systems Ltd.). Absorbance was calculated by following the methodology of Kinney et al., 2000.^37^ The SSR method assumes that PM_1_ is evenly distributed on the filters. The Sioutas impactor occasionally concentrates the impaction of PM into the center of the final filter (PM_1_), thereby adding uncertainties to the BS measurements. These filters (*n* = 2) were omitted from BS analysis.

### Polycyclic Aromatic Hydrocarbons

PAHs were sampled at 18 restaurants following NIOSH Method 5515 ^38^ and analyzed using the updated NIOSH Method 5528 ^39^ for lower detection limits and specificity. PM-bound and gaseous PAHs were captured on the previously described 37 mm PTFE filter and an 8 × 110 mm sorbent tube (226–30–05, SKC)—with a primary glass wool plug, a primary XAD-2 sorbent, followed by both a secondary glass wool and XAD-2 section—attached to an AirChek XR5000 (SKC) sampling pump operating at 2 L/min in tandem. After sampling, the media were promptly covered in aluminum foil and kept at −20 °C to minimize losses until analysis.

For speciation and quantification, the PTFE filter, primary glass wool plug, and primary XAD-2 sorbent were separated from the secondary glass wool plug and sorbent. The primary and secondary sections were placed in separate amber vials. Analytes were then extracted using 2.0 mL of HPLC grade methylene chloride (75–09–2, Thermo Scientific) by mixing well and placing in an ultrasonic water bath for 30 min. Resulting extracts were filtered using 0.45 μm Acrodisc PTFE syringe filters (Pall) and subsequently diluted 10-fold with methylene chloride. Dilution was required due to the higher sensitivity of the MS instrument, compared to the one utilized in method no. 5528. Aliquots (200 μL) of the diluted extracts were placed in a GC-vial. These were spiked with 1 μg internal standards using EPA 8270 Semivolatile Internal Standard Mix (CRM46955, Sigma-Aldrich).

Samples were analyzed using gas chromatography–mass spectrometry (GC/MS) at the NYU Grossman School of Medicine. A JMS-TQ4000 (JEOL Ltd.), coupled with 8890GC System (Agilent Technologies), was used for the identification and quantification of 16 PAHs (Table S2) using a selected ion monitoring (SIM) acquisition method. The system utilized a 30 m × 0.25 mm, J&W HP-5 ms GC column (Agilent) for separating the target compounds.

For each organic analyte, calibration curves were prepared using an EPA 8310 polynuclear aromatic hydrocarbon mix (CRM47543, Sigma-Aldrich) to obtain six working standards with the following concentrations: 0.0125, 0.0375, 0.125, 0.375, 1.25, and 3.75 μg/mL. All calibrations were then spiked with 1 μg/mL of the EPA 8270 Semivolatile Internal Standard Mix (CRM46955, Sigma-Aldrich). Target compound calibration curves were found to follow a quadratic regression model, as described in the 5528 protocol, except for naphthalene, which fit better with linear regression. All calibration points achieved accuracy within 20%, with regression coefficients *R* ^2^ exceeding 0.99. The samples were analyzed in three batches, where each batch contained two quality control vials and two method blanks that were injected for quality assurance throughout the run. Method blanks showed trace responses. Most (15/16) analyses passed the recovery test for control samples (spike level at 25 μg/sample), with an acceptance range of 75–125%. The relative standard deviation (RSD) for control samples was below 20%. All sample values were obtained by subtracting the average level measured in blanks.

### Volatile Organic Compounds

VOC speciation for C1 to C10 compounds, followed NIOSH Method 3900,^40^ and the chemical analysis was performed at the National Institute for Occupational Safety and Health (NIOSH) Field Studies Branch Organics Laboratory in Morgantown, WV (the “NIOSH lab”). All canister sampling components were prepared by the NIOSH lab and shipped to NYUGSoM in batches consisting of 4 canisters (3 for air sampling and 1 field blank). Evacuated 450 mL Silonite-coated MiniCans (“canisters”) (29-MC450SQT, Entech Instruments, Inc.) were used to obtain 8 h time-integrated samples exclusively in the kitchen area using a restrictive flow controller operating at 0.3 mL/min. Prior to each deployment, canisters were cleaned and evacuated using an Entech Instruments 3100D Canister Cleaning System following EPA Compendium Method TO-15.^41^ Flow controllers were also cleaned using UHP nitrogen and assessed for flow accuracy prior to deployment. Additionally, the vacuum level in each canister was confirmed by attaching a MicroValve vacuum test gauge (01–29–70010QT, Entech Instruments, Inc.). After sampling, the flow controllers were removed, and the canisters were stored at room temperature for up to 30 days prior to analysis, reducing sample loss due to sample instability.^42^ The parts per billion (ppb) concentrations of 18 VOCs were then analyzed using a 7890*B*/5977A GC/MS (Agilent Technologies).

### Statistical Analyses

Statistical analyses were performed using RStudio (v.2023.06.2) with R (v.4.5.0) software. Figures were created using the software and with GraphPad Prism (v.9.5.0). Briefly, a Mann–Whitney *U* test was performed on nonparametric PM concentration data to determine significant differences (*p* ≤ 0.05) where normality was determined by Q–Q plot residuals. Confidence intervals (95% CI) were determined through bootstrapping.

To further assess the relationship between PM_2.5_ concentrations in kitchens and dining areas across restaurants (Figure S1), and whether this relationship was moderated by restaurant layout (open vs closed), we fitted a linear mixed-effects model. An open-layout restaurant was defined by having kitchens that were visible to diners, with no physical barrier to the exchange of air between areas. The linear mixed-effects model was fit with fixed effects for scaled kitchen PM, layout (open vs closed), and their interaction. Restaurant ID was included as a random intercept and random slope for kitchen PM, with intercept–slope covariance constrained to zero. Additionally, potential variables impacting PM_2.5_ exposures—such as “distance from the exhaust hood” and “day of the week” effects—were investigated in separate sensitivity analyses (Figure S2).

Correlations between different pollutant concentrations were determined by a Kendall-Tau correlation matrix. Relationships between categorical data (restaurant average price range, neighborhood income, seasonality) and average pollutant concentrations (VOCs and PM) were explored using Kruskal–Wallis Tests with a Holm-Bonferroni correction (Table S1). Exploratory factor analysis (EFA) with oblique rotation was conducted on VOC and PM XRF data to identify latent groupings of compounds. The number of factors retained was determined based on visual inspection of the scree plot (Figure S3), identification of the eigenvalue “elbow,” and minimization of the extended Bayesian Information Criterion (eBIC). Factor interpretability was also considered.

## Results and Discussion

### Particulate Matter Concentration and Composition

Kitchen areas had a median (Md) PM_2.5_ concentration of 79.6 μg/m^3^, whereas dining areas had a median concentration of 20.0 μg/m^3^, and outdoor ambient concentration was 8.4 μg/m^3^. One kitchen sample was lost; thus, 17 kitchen samples were compared to the 18 dining areas samples. Gravimetrically measured PM_2.5_ concentrations from 18 restaurants differed significantly between areas. The 95% confidence intervals for the differences were 7.1–74.0 μg/m^3^ (kitchen vs dining; *p* = 0.003), 20.5–81.9 μg/m^3^ (kitchen vs outdoor; *p* < 0.0001), and 4.9–21.1 μg/m^3^ (dining vs outdoor; *p* < 0.001) (Figure 2). Similarly, PM_10_ concentrations were significantly higher in kitchens (Md = 86.6 μg/m^3^) compared to dining areas (Md = 22.4 μg/m^3^; 95% CI: 11.3–80.3, *p* = 0.002). PM_1_ on average accounted for approximately 74% of the PM_10_ mass found in the kitchens, and 68% in the dining areas. PM_2.5_ accounted for about 84% and 81% of PM_10_ mass, respectively. Four kitchens had gravimetric PM levels between 4 and 10× higher than the median (outliers) Figure 2.

Minute-by-minute aethalometer measurements indicated higher BC concentrations in kitchens compared to dining areas; however, instrument noise and occasional negative values limited confidence in these data. Therefore, black smoke (BS) absorbance measured on PM_1_ filters is presented as the primary indicator of carbonaceous particles. Overall, BS absorbance failed to demonstrate a significant difference between kitchens and dining areas (*p* = 0.062; Table 1).

**Table 1:** Median Mass Concentrations (ng/m^3^) and Ranges for Kitchen and Dining Room Areas of PM_1_, Black Smoke Absorbance (abs), and Particle-Bound Elements^a^

		kitchen			dining room		
analyte	LOD	*n*	median	IQR	*n*	median	IQR
**PM**_**1**_(μg/m^3^)	0.88	17	**73.6**	150.7	18	**15.8**	23.8
abs (10^–5^/m)	-	17	7.34	13.7	16	3.83	4.84
sodium (Na)	-	-	<LOD	-	-	<LOD	-
magnesium (Mg)	28.7	-	<LOD	-	-	<LOD	-
aluminum (Al)	-	-	<LOD	-	-	<LOD	-
silicon (Si)	26.6	17	157	208	18	176	217
phosphorus (P)	-	-	<LOD	-	-	<LOD	-
sulfur (S)	5.71	17	441	310	18	255	342
**chlorine (Cl)**	22.3	17	**283**	470	18	**82**	157
**potassium (K)**	5.36	17	**169**	390	18	**69.2**	76.4
calcium (Ca)	20.7	17	53.5	52.6	18	57.5	80.1
titanium (Ti)	3.81	17	7.81	15.8	18	7.72	7.76
vanadium (V)	0.231	17	0.15	0.65	18	0.25	0.36
chromium (Cr)	0.49	17	0.98	0.72	18	1.01	0.63
manganese (Mn)	0.39	17	2.63	3.3	18	2.25	2.91
iron (Fe)	7.94	17	69.6	95.6	18	80.5	107
cobalt (Co)	0.055	17	0.1	1.13	18	0.53	1.32
nickle (Ni)	-	-	<LOD	-	-	<LOD	-
**copper (Cu)**	**0.80**	**17**	**4.92**	**4.44**	**18**	**2.72**	**4.02**
zinc (Zn)	5.28	17	12.3	9.58	18	10.8	18.1
arsenic (As)	0.32	17	0.16	0.3	18	0.16	0
selenium (Se)	0.16	17	0.08	0.28	18	0.15	0.24
lead (Pb)	1.36	17	2.3	2.05	18	0.7	2.06

a Abbreviations: LOD, limit of detection; IQR, interquartile range; *n* = the number of samples analyzed. Concentrations in ng/m^3^ unless otherwise stated. Significant differences noted in bold.

We note that kitchen BC concentrations from the aethalometer were strongly correlated with BS absorbance (*r* ^2^ = 0.84), supporting consistency between methods in kitchens, whereas the correlation was weak in dining areas (*r* ^2^ = 0.1). Mean kitchen BC concentrations from the aethalometer were 2.6 μg/m^3^. Although this value should be considered an approximate estimate, its magnitude is comparable to previously reported indoor BC concentrations in NYC apartments (∼3 μg/m^3^), suggesting that combustion-related carbonaceous particle levels in restaurant kitchens may fall within the range observed in other urban indoor environments.

XRF analysis of gravimetric PM_1_ samples revealed that the speciated trace elements composed an average of 4.5% of the mass of kitchen particles, whereas total mass reconstructed as commonoxides^43^ was 5.0%. Chlorine, potassium, and copper were found in significantly higher (*p* ≤ 0.05) concentrations in the kitchen. For dining room samples, trace elements composed 5.7% of the PM_1_ mass and 6.4% when converted to common oxides. In the previously mentioned indoor experiments, metals composed between 8 and 10% of PM_2.5_ average mass.^44^

A particle’s toxicity is often informed by both its size and composition. The elemental content of the aerosols in the dining room slightly differs from those in the kitchen, although on average, Cl, K, and Cu were found in higher concentrations in the kitchen samples (Table 1). These differences may reflect the use of cleaning agents (K, Cl), biomass combustion (K, Cl) and meat cooking (Cu) in the kitchen area.^45^ Exploratory factor analysis partially supports this theory; each area is made of three factors, or elemental thumbprints (Table S3), with the kitchen having a cooking emissions factor (Ca, Fe, Mn), a combustion factor (S, Se, V), and a distinct metals factor (Cu, Zn, Pb). A review paper from Sagara et al. 2023 has reported several studies linking Ca, Mn, and Fe, among other metals and organic components, to indoor cooking.^45,46^ The second factor (S, Se, V) is more characteristic of fossil fuel combustion tracers. Thurston et al. 2011 has attributed V and Se as markers of residual oil and coal combustion through atmospheric source apportionment.^47^ As charcoal combustion was not observed within the restaurants, this factor likely reflects infiltration of outdoor combustion emissions from either traffic or oil-fired heating systems. Lastly, we posit this grouping may reflect outdoor infiltration of particles from road dust, for which Pb and Cu are common tracers from car brake wear.^48^ The unique kitchen metals factor might also represent metals released during scraping of pans, spatulas, and other metal abrasions. Cu and Zn have also been associated with cooking emissions and mechanical wear of kitchen equipment; however, attributing Pb to cookware abrasion is unlikely, as cookware is not expected to be a substantial lead source.^45^ The first dining room factor is more mixed (Cu, Pb, As, Si, Cl). This mixed dining room metals factor reflects indoor penetration of outdoor traffic/resuspension particles or mixed cooking residues.^47^ The second metals factor (Ca and Fe) is like the cooking emissions factor seen in the kitchen particles. Lastly, the third factor (S and Se) likely indicates particle intrusion from outdoor combustion sources, coal or oil, or from gas combustion in the kitchen. These data are exploratory in nature but agree with previous source apportionment research conducted in NYC residences by Habre et al. 2014.^49^ These data also suggest particle intrusion from the kitchen into the dining areas based on the metallic differences, thus suggesting insufficient ventilation in the cooking zone.

Notably, the composition of metals in the particles from both areas was negligible compared to the organic, ionic, and carbonaceous mass fraction. XRF only explains about 5.0% of the mass on kitchen filters, indicating that the restaurant PM is principally carbon-based (Figure S4). The degradation of fats, proteins, and carbohydrates—in which the wonderful flavors and aromas we associate with cooking are produced—could offer an explanation.^50^ The thousands of new compounds produced in Maillard reaction, sugar caramelization, fermentation, or oxidation are aerosolized by cooking activities. Thus, due to their organic nature they remain undetected by XRF. Indoor air pollution research has shown that black carbon, secondary aerosols, nitrates, and sulfates also contribute to this fraction.^49^ Past experiments have detected aerosolized fat-soluble molecules, aldehydes, and ketones. We recommend future experiments to consider these and other particle-bound toxicants with nontargeted mass spectrometry methods to inform toxic potential of these particles.

### PAH Concentrations

Whereas most PAHs concentrations were below the LOD (518 out of 560 samples) in the 18 restaurants, some analytes were still quantified at low levels. Naphthalene was detected in most kitchens (*n* = 14), with a median concentration of 145.8 ng/m^3^ [IQR: 117.3–232.4], and dining rooms (*n* = 14; Md = 145.4 ng/m^3^, IQR: 110.1–178.4) (where *n* is the number > LOD). Only two restaurant kitchens had values of other PAHs above LOD, which correspond to the kitchens with outlying PM concentrations (Figure 2).

PAHs detected above the LOD were found in concentrations comparable to what has been reported in Western-style commercial kitchens. As the median concentrations for naphthalene were similar for kitchens and dining rooms, with no correlation to PM concentrations for their respective restaurants, we posit these levels reflect background values.^51^ However, it is the relative lack of PAH concentrations above the LOD, across the sampled restaurants, that may offer some exploratory information regarding PAHs in the restaurant industry. We suggest that the Western-style restaurant environment, both kitchen and dining areas, may be naturally low in PAHs, particularly when compared to their Eastern restaurant counterparts. Eastern-style cooking operates at higher temperatures and requires significant physical stirring and tossing, generating large amounts of PM and aerosolized oil droplets.^52^ Additionally, wok hei—the charred aroma associated with Cantonese cooking—imbues a smokiness to its food achievable only by intense heat and singeing oil, thus inevitably generating PAHs. Since Western-style cooking is less focused on smoky flavors and requires more moderate temperatures, lower concentrations of PAHs are produced in general, as evidenced by prior research, and these may be removed by exhaust hoods before permeating further into the kitchen area.^53^ Fire codes state that grease or smoke producing appliances in commercial kitchens must be exhausted by a ventilation system, with the NYC Mechanical Code specifying a type I hood. Compliance in NYC is carried out through annual inspections, suggesting strict adherence.^54^ Therefore, our sampling methodology may have failed to capture the low levels of kitchen PAHs before they were exhausted. Wood burning was observed in two kitchens (Table S1), and while a known generator of PAHs,^55^ these kitchens did not yield concentrations above the LOD. Additionally, while high temperature frying has been shown to generate PAHs, they were not found in appreciable amounts in the sampling areas. Despite these observations, previous literature has shown that Western-style cooking activities can generate PAHs, but measurements were often conducted near the cooking activity or by personal sampling.^6,22,56^

Samples were collected on a combination of Teflon filter and XAD-2 sorbent tube. Updated NIOSH methods have used the XAD-7 sorbent for PAH collection, which may confer slight advantages in vaporized PAH collection. However, these advantages might be minimal, since extremely little, if any, PAHs were detected in the breakthrough portion of the XAD-2 tube. Additionally, the sampling volume in our method was doubled to account for these differences.

### VOC Concentrations and Composition

VOCs were generally detected in amounts that seldom exceeded 1000 ppb and did not exceed applicable NIOSH recommended exposure limits (RELs).^57^ However, compounds such as ethanol and isopropanol were found in nearly every restaurant kitchen, appearing in at least 95% of samples (Table 2). Toluene and chloroform were detected in about half of all samples. Benzene was detected in about one-third of samples; however, measured concentrations remained below the NIOSH REL of 0.1 ppm (8 h TWA). 2,3-Butanedione, 2,3-hexanedione, 2,3-pentanedione, ethylbenzene, styrene, *m*,*p*-xylene, or *o*-xylene were not detected in any sample. Lastly, NYC sets CO ceiling limits at 9.0 ppm (ppm), and this study did not detect any 8 h average concentrations above 2.0 ppm, nor short-term exceedances above the World Health Organization (WHO) guidelines of 2010 for CO.^58,59^

**Table 2:** Descriptive Statistics of 12 Detected Kitchen-Borne VOCs (2,3-Butanedione, 2,3-Hexanedione, 2,3-Pentanedione, Ethylbenzene, Styrene, *m*,*p*-Xylene, or *o*-Xylene Omitted for Clarity) Collected from 19 Samples (One Restaurant was Repeated)^a^

analyte	LOD (ppb)	*n*	median (ppb)	IQR (ppb)	maximum (ppb)
ethanol	0.27	19	730.4	877.9	18987.7
isopropyl alcohol	0.24	18	26.7	196.8	4262.4
acetone	0.21	14	14.5	26.2	285.0
chloroform	0.21	9	0.0	1.5	2.9
toluene	0.21	9	0.0	2.2	103.3
d-limonene	0.26	7	0.0	3.7	22.5
benzene	0.21	6	0.0	1.1	2.2
acetonitrile	0.28	3	0.0	0.0	2.8
α-pinene	0.27	1	-	-	2.8
methylene chloride	0.21	1	-	-	1.4
methyl methacrylate	0.21	1	-	-	1.5
n-hexane	0.25	1	-	-	107.6

a Abbreviations: LOD, limit of detection; ppb: parts per billion; IQR, interquartile range; *n* = the number of samples detected above LOD.

Exploratory Factor Analysis (Figure S5) of the VOC components produced three loadings where components strongly varied together. Using a correlation matrix, kitchen pollutants generally had slight to moderate relationships with any pollutant, whether positive or negative (Figure S6). VOCs such as benzene, ethanol, isopropanol, and d-limonene often had no relationships with PM, BC, or BS. Kitchens occasionally had outlying concentrations of individual pollutants that did not necessarily correlate with other VOCs (i.e., one kitchen had methyl methacrylate and no other remarkable pollutants). We offer multiple hypotheses that could explain such a dynamic environment based on previous literature.

The most likely source of recurrent VOCs are cleaning agents. Recent academic literature on usage of specific cleaning agents is sparse, yet these among blog posts, advertisements, and health standards help in creating holistic pictures. Cleaners often consist of sanitizing agents, dish soap/detergent, degreasers, stainless steel polish, and specialized food service chemicals.^60–62^ Factor analysis (Figure S5) on VOC concentrations yielded three distinct factors, giving an understanding to these sources. Factor 1, composed of *n*-hexane, toluene, and acetone indicates the use of a solvent based cleaner in the kitchen area. This mixture of polar and nonpolar compounds enables grease to be solubilized quickly and broken down. Stainless steel cleaners or degreasers often contain acetone and mixed hydrocarbons, but their composition can vary by manufacturer.^63–65^ Similarly, Factor 2 is likely a cleaning soap with citrus scent—d-limonene and ethanol are highly correlated in this loading. Anecdotally, chefs and kitchen managers indicated that most kitchens use very similar bulk, industrial grade cleaners and do not often venture out into specialty products when soap and water might be best. Lastly, factor 3 is indicative of regular kitchen activities. While it may not represent cooking processes, benzene is off-loaded in this factor; it is released from natural gas burners when cooking is occurring.^11^ This inverse relationship may be saying that when cooking is occurring, cleaning is likely not, and vice versa.^66^ Isopropyl alcohol containing products are typical for sanitizers.^67,68^

### Limitations

It is imperative to note, by nature of the cascade impactor, PM_1_ was collected on the 37 mm filters. To adhere to previously established analytical techniques, these 37 mm filters were used for analysis of metallic PM constituents. In this study, PM_1_ on average accounted for about 92% and 72% of PM_2.5_ mass with respect to the kitchen and dining areas; therefore, these PM_1_ measurements may underestimate the concentrations of the XRF detected elements and BS in fine mode. Additionally, OSHA PELs and NIOSH RELs are intended to be directly compared with personal full-shift measurements; therefore, area air samples are only an indication of potential personal exposure and approximate true exposures. Despite these limitations, the cascade impactor was essential for area sampling in this study to characterize the thoracic and respirable portions of an unknown environment. Respirable dust standards set by the OSHA consider a PM_4_ cut point,^57^ while evidence suggests smaller fractions of PM impacting in the alveolar region.^69–71^ Additionally, epidemiological evidence links fine particles with cardiovascular mortality and systemic diseases.^72^ The implementation of real-time and integrated PM_2.5_ measurements in this study better captures the fraction of PM that imposes the greatest health concerns.

VOCs were exclusively quantified in the kitchen area due to the limited number of canisters available. Therefore, it is difficult to determine whether all VOCs originated from kitchen activities. Cleaning occurs in all parts of the restaurant, and air exchange or off gassing could influence the measured concentrations. Furthermore, using EFA with a limited sample size may reduce the stability of the loadings; having a larger sample size in future studies would strengthen these conclusions.

Another limitation stems from the types of chemicals analyzed; NIOSH method 3900 has been often used in occupational healthcare settings and excels in low molecular weight identifying solvents. This was advantageous in our study because of the industrial cleaners often present in this environment, and these compounds have been seldom quantified in restaurants prior to this experiment.^66^ Alternatively, this study could have been strengthened by analyzing chlorinated compounds, NO_x_, or SO_x_, which can modify particle toxicity and are implicated in overall health outcomes. Previous literature has indicated that these air pollutants, among other hydrocarbons, are present in the kitchen environment and necessitate investigation.

The modest number of restaurants, single metropolitan focus, and selective chemical scope (e.g., VOCs only in kitchens; exclusion of aldehydes, ketones, NO_x_, SO_x_) could mean these findings underestimate or incompletely represent exposures across industry. Other studies have shown that repeated measurements at the same restaurant (Figure S2) can yield substantially different concentrations.^73^ The meals sampled (i.e., breakfast and lunch vs lunch and dinner) may differ in emissions. We recognize the difficulty of interpreting these results with a small sample size. Thus, we hesitate to generalize these findings to the rest of the restaurant industry; however, findings from this study provide a preview into the current state of restaurants in New York City. In future studies, a larger number of restaurants studied could enable better resolutions for price index, seasonality, layout, day-of-the-week, and sampling location patterns. Information on ventilation efficiency, air exchange rates, meal output, staff size, and dimensions of the restaurant can add critical context in explaining these aerosol concentrations. Moreover, using PM_2.5_ rather than PM_1_ for speciation and accounting for variables such as pressure, temperature, and relative humidity differentials could yield results that better elucidate the relationship between the restaurant kitchen and dining room (Figure S1).

### Health Implications

In all cases, PM concentrations were less than the OSHA permissible exposure limit of 5 mg/m^3^ for respirable particles.^57^ However, median concentrations of PM_2.5_, in the kitchens and dining areas were well above the 2021 WHO air quality guidelines, i.e., 24 h average exposures should not exceed 15 μg/m^3^ more than 3–4 days per year.^59^ While the WHO guidelines are applicable to indoor environments, they do not cover occupational settings due to the specific characteristics and unique exposures in the workplace. However, they may be used as a frame of reference for understanding induced health effects. Nevertheless, both kitchen staff (e.g., chefs, cooks, dishwashers, etc.) and dining room staff (e.g., maître di, servers, bartenders, etc.) are exposed to high concentrations of respirable particles with distinct compositions. These particles are likely composed of decomposed organic molecules, combustion products, and mutagenic aldehydes with uncharacterized toxicities. In addition, the quick spikes in PM_2.5_ may also contribute to acute health impacts such as wheeze or cough. The chronic exposures to low levels of VOCs may also be cause for concern. Moreover, our measurements likely underestimate the true exposures for kitchen workers, whose jobs involve moving between and around the cooking and prep stations.

Lastly, we note what is already well-known to restaurant staff; this environment—with a documented history of high temperatures, loud noise levels, and longer working hours leading to longer exposures—could be dangerous for the long-term health of its employees. These results, plus emerging air pollution data, paint a concerning picture of the exposome for restaurant staff, particularly kitchen workers. Future research should prioritize health assessment of restaurant staff, expanded chemical characterization of particle-bound organics (e.g., aldehydes, ketones), comparisons between natural gas and electric cooking, and intervention studies evaluating the effectiveness of filtration or engineering controls. These results highlight an understudied occupational health risk that warrants further investigation and protection for restaurant workers.

## Supplementary Material



## Notes

Additional PM concentration data and spatial saturation testing results: Figures S1–S2; additional VOC and speciation data regarding factor loadings and correlation analysis: Figures S3–S6; additional characteristics of restaurants, PAHs, and EFA loadings: Tables S1–S3 (PDF)
