# Supplementary material for: Occupational Exposures in the Culinary Underbelly: Air Pollution in Restaurants
Source: Environ Sci Technol. 2026 Jun 21;60(26):18723–32. doi: 10.1021/acs.est.5c18025 (PMC13348163; doi:10.1021/acs.est.5c18025)
Supplement: Supplementary file 1 [file es5c18025_si_001.pdf]

Title
Occupational exposures in the *Culinary Underbelly*: Air pollution in restaurants

Author Block
Antonio F. Saporito[1*], Mehmood Hashmi[1], Wuyue Yu[1], Drew R. Michanowicz[2], Eric D. Lebel[2], Nicole Lucha[2], Gan Huang[2], Colin J. Finnegan[3], Vittorio Albergamo[4], Wenjie Lyu[4], Steven N. Chillrud[5], James M. Ross[5], Dru A. Burns[6], Ryan F. LeBouf[6], Christopher Galarza[7], Judith T. Zelikoff[1], Terry Gordon[1]

[1]Division of Environmental Medicine, NYU Grossman School of Medicine, New York, NY, 10010, USA
[2]PSE Healthy Energy, Oakland, CA, 94612, USA
[3]Department of Earth System Science, Stanford University, Palo Alto, CA, 94305, USA
[4]Department of Pediatrics, NYU Grossman School of Medicine, New York, NY, 10016, USA
[5]Lamont-Doherty Earth Observatory, Columbia University, Palisades, NY, 10964, USA
[6]Respiratory Health Division, National Institute for Occupational Safety and Health, Morgantown, WV, 26508, USA
[7]Forward Dining Solutions LLC | EcoChef, Pittsburg, PA, 15147, USA

*Antonio F. Saporito
(856)-472-2134
Antonio.saporito@nyulangone.org
341 East 25th St
NYU Grossman School of Medicine
NY, NY 10010

Summary
Pages: 12; Figures: 6; Tables: 3

Table S1. Characteristics of restaurants sampled.

ID	Sampling Date	Hours Sampled	Price Point	NYC Borough	Cooking Fuel	Cuisine Type	Cooking Techniques
1	12/08/23	0800-1600	$20–30	Manhattan	Natural Gas	American	Grilling, Sautéing, Roasting, Deep-frying, baking
2	12/16/23	1000-1800	$30–50	Brooklyn	Natural Gas	Southern / Soul	Deep-frying, Grilling, Sautéing, Roasting, stewing
3	12/19/23	1000-1800	$20–30	Brooklyn	Natural Gas & Wood	Modern American	Grilling, Sautéing, Roasting, stewing, wood fired
4	01/23/24	1000-1800	$10–20	Brooklyn	Natural Gas	American	Roasting, Deep-frying, Grilling, Sautéing, baking
5	02/07/24	0800-1600	$10–20	Brooklyn	Natural Gas	American Diner	Deep-frying, Grilling, Sautéing, Roasting, stewing
6	02/09/24	0900-1700	$10–20	Brooklyn	Natural Gas	Latin Diner	Deep-frying, Grilling, Sautéing, Roasting, stewing
7	03/06/24	1100-1900	$30–50	Manhattan	Natural Gas	American	Deep-frying, Grilling, Sautéing, Roasting,
8	04/03/24	0800-1600	$10–20	Brooklyn	Natural Gas	American Diner	Deep-frying, Grilling, Sautéing, Roasting, Stewing
9	04/05/24	0800-1600	$10–20	Brooklyn	Natural Gas	American Diner	Deep-frying, Grilling, Sautéing, Roasting, stewing
10	07/25/24	0800-1600	$10–20	Manhattan	Natural Gas	American	Deep-frying, Grilling, Roasting, stewing, baking
11	08/01/24	1200-2000	$20–30	Brooklyn	Natural Gas	Burger / American	Deep-frying, Grilling, Sautéing, Roasting
12	08/15/24	1230-2030	$20–30	Brooklyn	Natural Gas	Burger / American	Deep-frying, Grilling, Sautéing, Roasting

13	10/01/24	0800-1600	\$20–30	Brooklyn	Natural Gas	American	Deep-frying, Grilling, Sautéing, Roasting, stewing, baking
14	10/03/24	1400-2200	\$100+	Manhattan	Natural Gas	Modern French	Deep-frying, Grilling, Sautéing, Roasting, stewing, baking
15	10/08/24	1400-2200	\$100+	Manhattan	Natural Gas	Modern French	Grilling, Sautéing, Roasting, Stewing, baking
16	11/20/24	1200-2000	\$10–20	Brooklyn	Natural Gas	Burger / American	Deep-frying, Grilling, Sautéing, Roasting
17	11/25/24	1200-2000	\$50-100	Manhattan	Electric, Wood, & Natural Gas	Modern American	Deep-frying, Grilling, Sautéing, Roasting, stewing, wood fired
18	01/18/25	1500-2300	\$100+	Brooklyn	Natural Gas	Modern French	Deep-frying, Grilling, Sautéing, Roasting, stewing, baking

When evaluating summer (June-August) and winter (December-February) months as a factor for the group of 18 restaurants, we found no significant difference ($p = 0.15$) between any sized PM concentrations in the dining area or the kitchen. However, when considering the NYC heating season (October 1st - May 31st) and non-heating season specifically, kitchen PM and dining room PM concentrations were significantly different ($p \leq 0.05$). This may be attributed to the excess particle production from boilers and indoor heating during the heating season. Many commercial buildings use oil or natural gas–fired boilers for heating. These combustion-based heating systems emit $PM_{2.5,}$ BC, and trace metals that can infiltrate indoor environments via ventilation systems, shared air shafts, or natural air exchange. Although we did not directly measure boiler emissions, building heating operations and subsequent PM emissions could partially explain the observed indoor contrasts. Lastly, categorical data including restaurant price range and neighborhood income had no significant correlations with any pollutant concentration.

Table S2. Polycyclic aromatic hydrocarbon (PAH) names and CAS Numbers

Compound Name	CAS Number
Acenaphthene	83-32-9
Acenaphthylene	208-96-8
Anthracene	120-12-7
Benz[a]anthracene	56-55-3
Benzo[b]fluoranthene	205-99-2
Benzo[k]fluoranthene	207-08-9
Benzo[ghi]perylene	191-24-2
Benzo[a]pyrene	50-32-8
Chrysene	218-01-9
Dibenz[a,h]anthracene	53-70-3
Fluoranthene	206-44-0
Fluorene	86-73-7
Indeno[1,2,3-cd]pyrene	193-39-5
Naphthalene	91-20-3
Phenanthrene	85-01-8
Pyrene	129-00-0

Table S3. Factor “Loadings” for the 3-Factor Kitchen and Dining solutions. Key signature tracers (>0.7) noted in bold.

	Kitchen Loadings			Dining Room Loadings		
Element	Crustal/ Metals	Metallic coatings	Fuel Combustion	Mixed Soil/ Dust/Metals	Crustal/ Metals	Fuel Combustion
Si	0.4	0.14	0.12	**1.02**	0.04	-0.21
S	-0.13	0.55	**0.7**	0.29	-0.01	**0.81**
Cl	0.56	0.56	-0.33	**1.04**	-0.19	-0.20
Ca	**0.93**	-0.04	-0.35	-0.01	**0.83**	-0.09
Ti	-0.03	-0.20	-0.04	-0.11	0.64	0.24
V	0.25	-0.09	0.17	-0.28	0.09	0.68
Cr	**0.7**	0.00	-0.05	0.10	0.32	-0.01
Mn	**0.85**	-0.02	-0.16	0.40	0.56	0.31
Fe	**0.98**	0.09	0.01	0.19	**0.89**	-0.01
Co	0.34	0.07	0.42	-0.11	0.04	0.27
Ni	0.6	0.12	-0.02	-0.21	0.52	-0.27
Cu	-0.15	**0.93**	-0.38	**1.03**	0.04	-0.13
Zn	0.19	**0.96**	0.01	0.37	0.33	0.34
As	-0.02	0.01	0.54	**0.86**	0.09	-0.13
Se	-0.24	-0.04	**0.79**	-0.01	-0.39	**0.99**
Pb	0.10	**0.72**	0.18	0.52	0.09	0.46

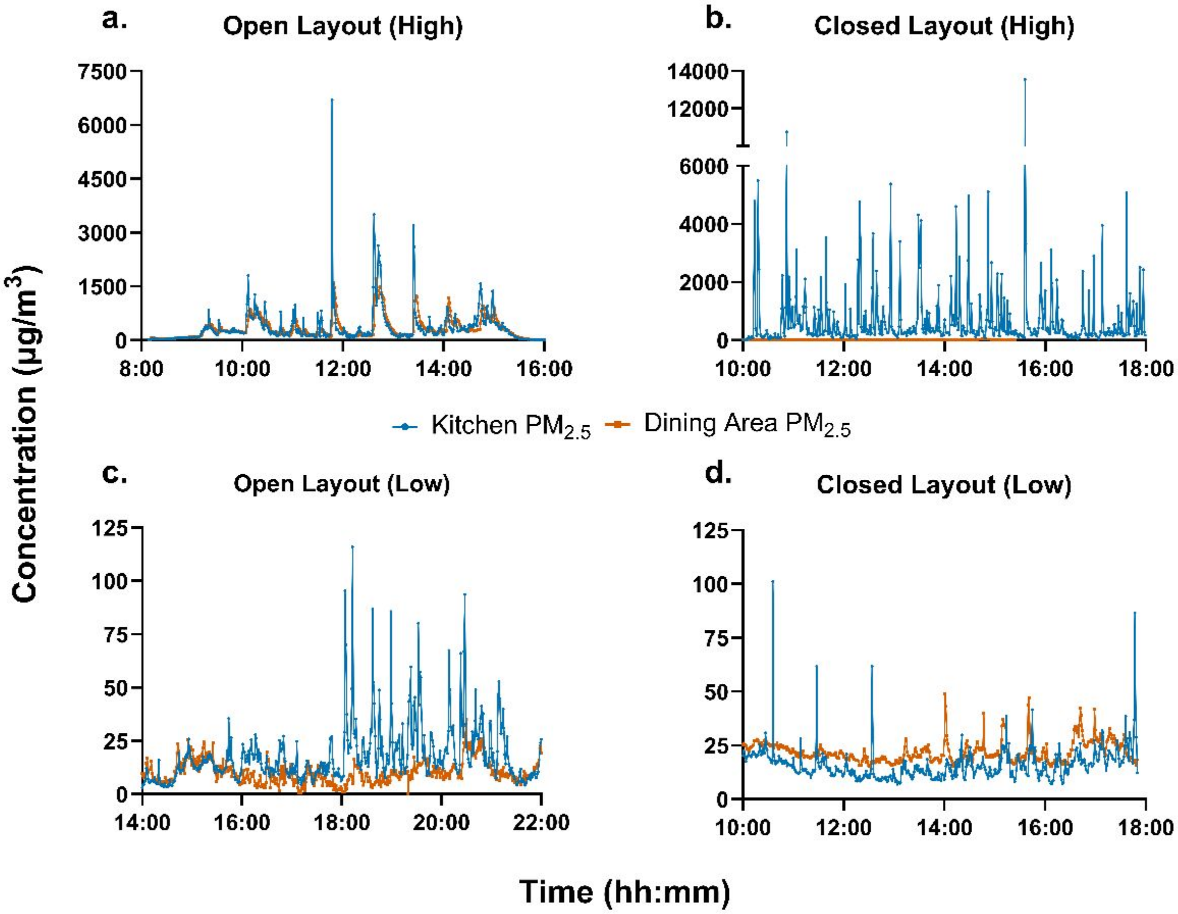

Figure S1(a-d). Two restaurants with high $PM_{2.5}$ concentrations (a,b) with open (a) and closed (b) layouts, along with two restaurants with low $PM_{2.5}$ concentrations (c,d) with open (c) and closed (d) layouts. Data are plotted at 1-minute intervals as recorded on a gravimetrically calibrated PDR-1500. 'High' and 'low' values are relative to the gravimetric median kitchen $PM_{2.5}$ concentration.

Real-time $PM_{2.5}$, BC, and CO data could indicate the potential for acute health impacts. Real-time $PM_{2.5}$ monitoring (Figure 3) demonstrated that concentrations of PM can spike into hundreds of $\mu g/m^3$ in minutes. Prior studies suggest that acute cardiovascular responses, including altered heart rate variability and blood pressure changes, can occur following $PM_{2.5}$ exposures over timescales ranging from minutes to hours. Real-time $PM_{2.5}$ data in Figure 3 exhibits a complicated relationship between open and closed concept restaurants. Open concept, where the kitchen and dining rooms are most often separated by counter space, would expectably allow for kitchen-borne $PM_{2.5}$ to influence the dining room concentrations (Figure 3a). Conversely, closed concept restaurants would expectedly not exhibit the same relationship due to the physical barrier between rooms. $PM_{2.5}$ at high concentrations in the kitchen did not always influence the concentrations in the dining area (Figure 3b). Unexpectedly, $PM_{2.5}$ in the dining area tended to peak with and follow similar concentration trends with the kitchen at low concentrations (Figure 3d). However, when analyzed using a mixed linear model, real-time data from the 18 restaurants

was not suggestive that open restaurant layouts significantly enabled kitchen-borne $PM_{2.5}$ to influence dining room pollution levels, despite high kitchen concentrations (Figure 3). ‘High’ and ‘low’ values are relative (above or below) to the median kitchen $PM_{2.5}$ in Figure 2. In closed-layout restaurants, a one SD increase in kitchen $PM_{2.5}$ was associated with a 24.8 µg/m^3 increase in dining $PM_{2.5}$ (SE = 50.7, t = 0.49). In open-layout restaurants, this effect was larger, with an estimated increase of 99.2 µg/m^3, yet also not statistically significant (t = 1.13). These data suggest the kitchen–dining PM relationship is much more complex. Random effects modeling indicated substantial variability across restaurants, indicating that other factors—not measured in this exploratory modeling—such as exhaust hood capture efficiency, kitchen size, dining room size, air change rate, and in-building ventilation impact the relationship. These aforementioned trends were not seen with real-time BC readings, nor did real-time BC readings correlate with real-time $PM_{2.5}$ concentrations likely due to the amount of noise in the BC data.

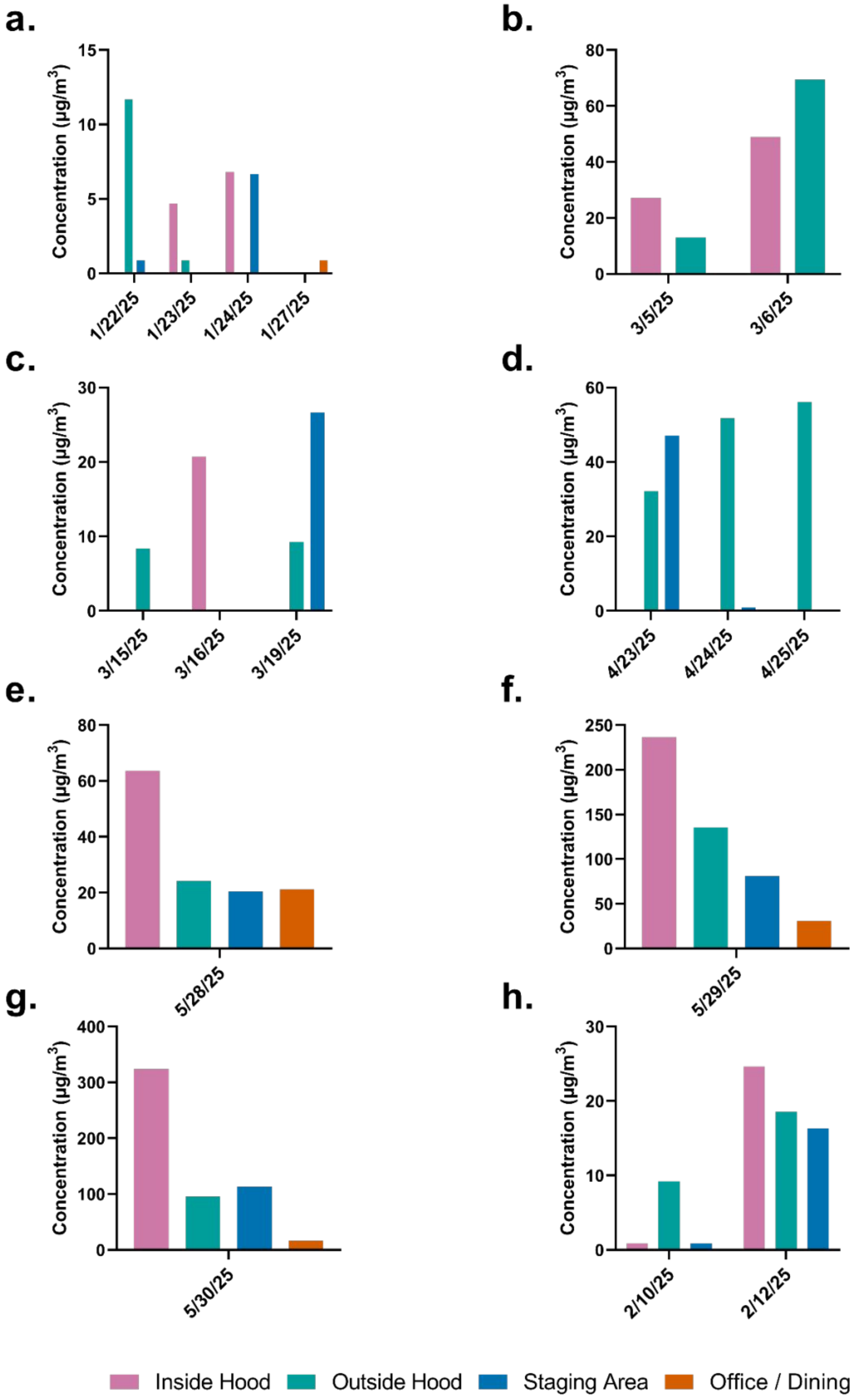

Figure S2(a-h). Concentrations of gravimetrically measured $PM_{2.5}$ depicting day-to-day variation and sampler placement location at eight restaurants.

Potential variables impacting $PM_{2.5}$ exposures—such as 'distance from the exhaust hood' and 'day of the week' effects—were investigated in separate sensitivity analyses. Eight additional restaurants, through a partnership with PSE Healthy Energy, were selected in the Bay Area, CA and Hudson River, NY. These results are considered separately from the main analysis due to the different sampling methodologies used in this collaboration. A subset of 37-mm PTFE filters and GK 2.05 cyclones (BGI) operated at 4 L/min on SKC pumps. All devices and materials were conditioned as described previously, and sampling was conducted for 8-hr periods. The cyclones were placed in one of four locations: in the exhaust hood, outside the exhaust hood, in the staging area, or in the dining/office area. These locations enabled us to capture the gradient of $PM_{2.5}$ concentration emanating from the stovetop, with maximum (in hood) and minimum (background) values. When possible, sampling was repeated multiple times within a week at the same restaurant to examine temporal effects on $PM_{2.5}$ emissions. Additionally, as kitchen staff's work is dynamic, locations were chosen to understand the integrated exposure to $PM_{2.5}$ experienced while cooking. For samples below the limit of detection, a concentration of 0.88 µg/m³ (LOD/2) was used in statistical analyses. We modeled log-transformed PM concentrations using a linear mixed-effects model (Equation 1) to understand the impact of day-to-day variation on PM concentration by sampling area. Fixed effects included sampling area ($\beta_1 \text{Area}_{ij}$), sampling day ($\beta_2 \text{Day}_{ij}$), and their interaction ($\beta_3 \text{Area}_{ij} \text{Day}_{ij}$). The interaction term assessed whether differences between kitchen and dining PM levels varied by day. Day was modeled as a categorical variable. A random intercept for restaurant (b_{0j}) accounted for repeated measurements within restaurants. The intercept (β_0) represents the expected log(PM) for the reference area on the reference day, and the residual term (ε_{ij}) represents unexplained within-restaurant variability. Random effects and residual errors were assumed to be normally distributed with mean zero.

$$\log(Y_{ij}) = \beta_0 + \beta_1 Area_{ij} + \beta_2 Day_{ij} + \beta_3 (Area_{ij} \times Day_{ij}) + b_{0j} + \varepsilon_{ij} \quad (1)$$

Potential variables impacting $PM_{2.5}$ exposure such as 'distance from the fume hood' and 'day of the week's effects' were investigated in eight restaurants sampled in the Bay Area, CA and Hudson River Valley, NY. Small, but not significant ($p = 0.12$), trends were seen while investigating the spatial distribution of $PM_{2.5}$ in the kitchen (Figure S3). Samples from inside the exhaust hood, with a median concentration of 25.9 µg/m^3 (IQR: [10.3–56.0]), were highest compared to outside the exhaust hood (21.3 µg/m^3; [9.9–55.1]), staging area (18.4 µg/m^3; [4.0–42.0]), or dining/office area (18.7 µg/m^3; [11.9–23.5]). We hypothesize these median concentrations, particularly the kitchen ones, are smaller compared to the median gravimetric $PM_{2.5}$ in NYC restaurants due to the larger kitchen sizes. The NYC kitchens were smaller in volume, likely leading to more concentrated PM. The effect of 'day' does differ numerically by sampling location (area), and indicate higher concentrations near the sources of pollution, but these effects are small. However, though not significant, the trend in median values could indicate that PM exposure is greater nearer to the cooking source, and pollution not vented through the exhaust hood disperses throughout the restaurant. These preliminary samples necessitate further investigation to confirm these patterns, but these data will be a useful indicator for future exposure studies.

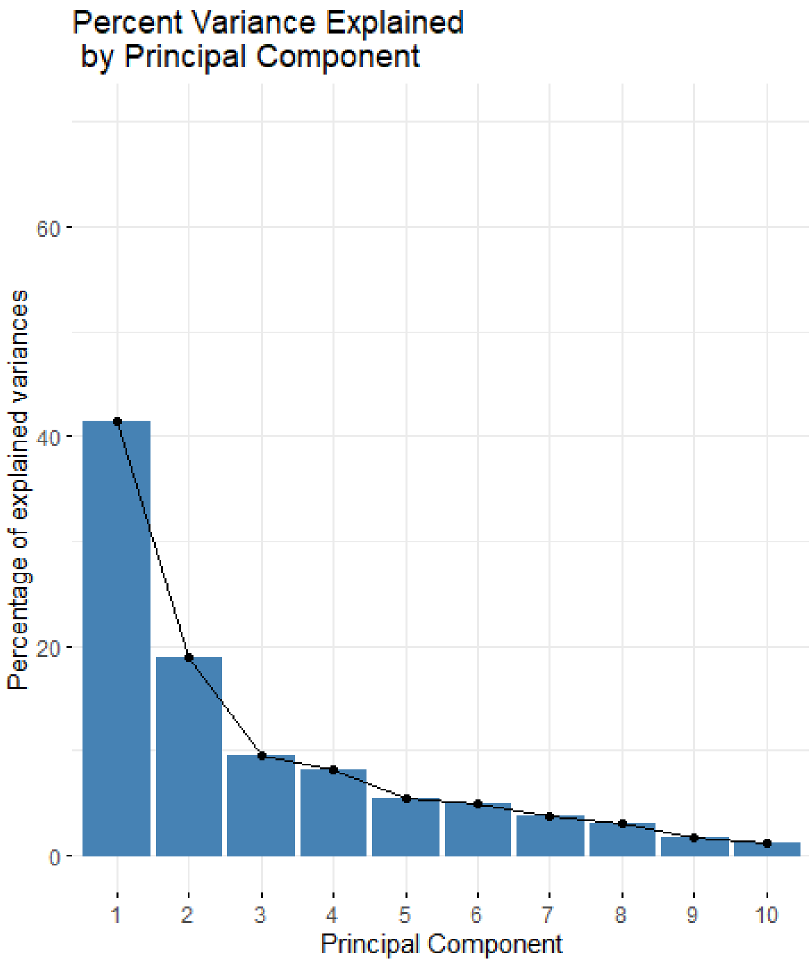

Figure S3(a). Scree plot explaining the percentage of variance from the factors from VOC analysis.

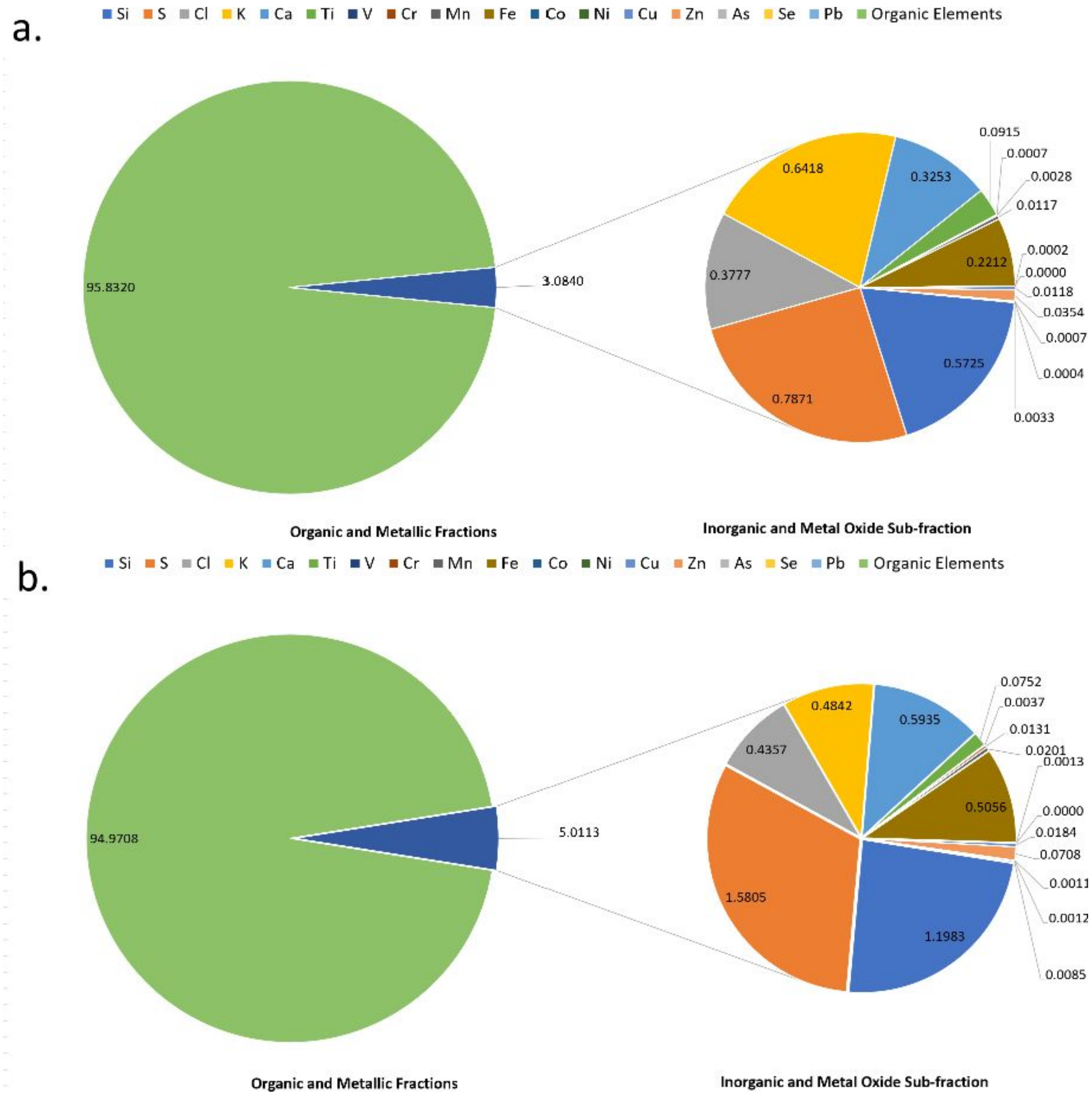

Figure S4(a,b). Median percent mass of PM_1 composition as determined by X-Ray Fluorescence (XRF) in both the kitchen (a) and dining areas (b) of 18 restaurants.

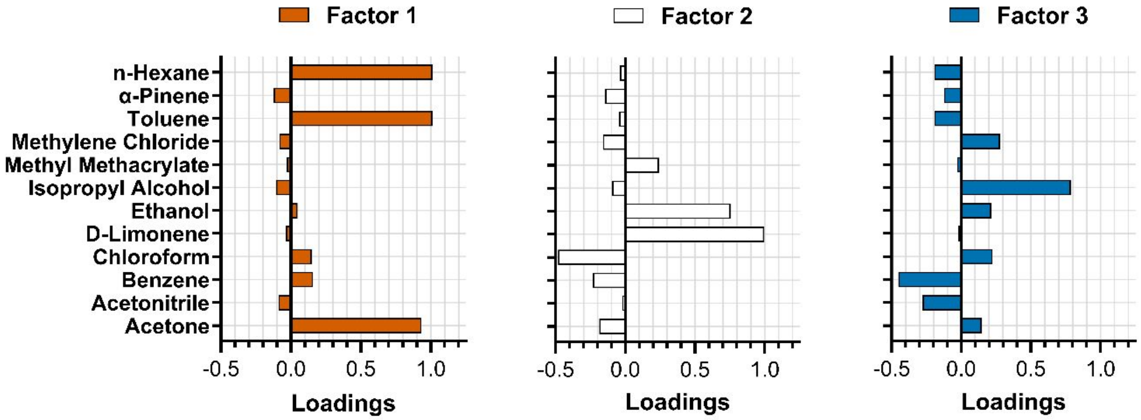

Figure S5. VOCs loadings from Exploratory Factor Analysis of 12 compounds detected in the kitchen.

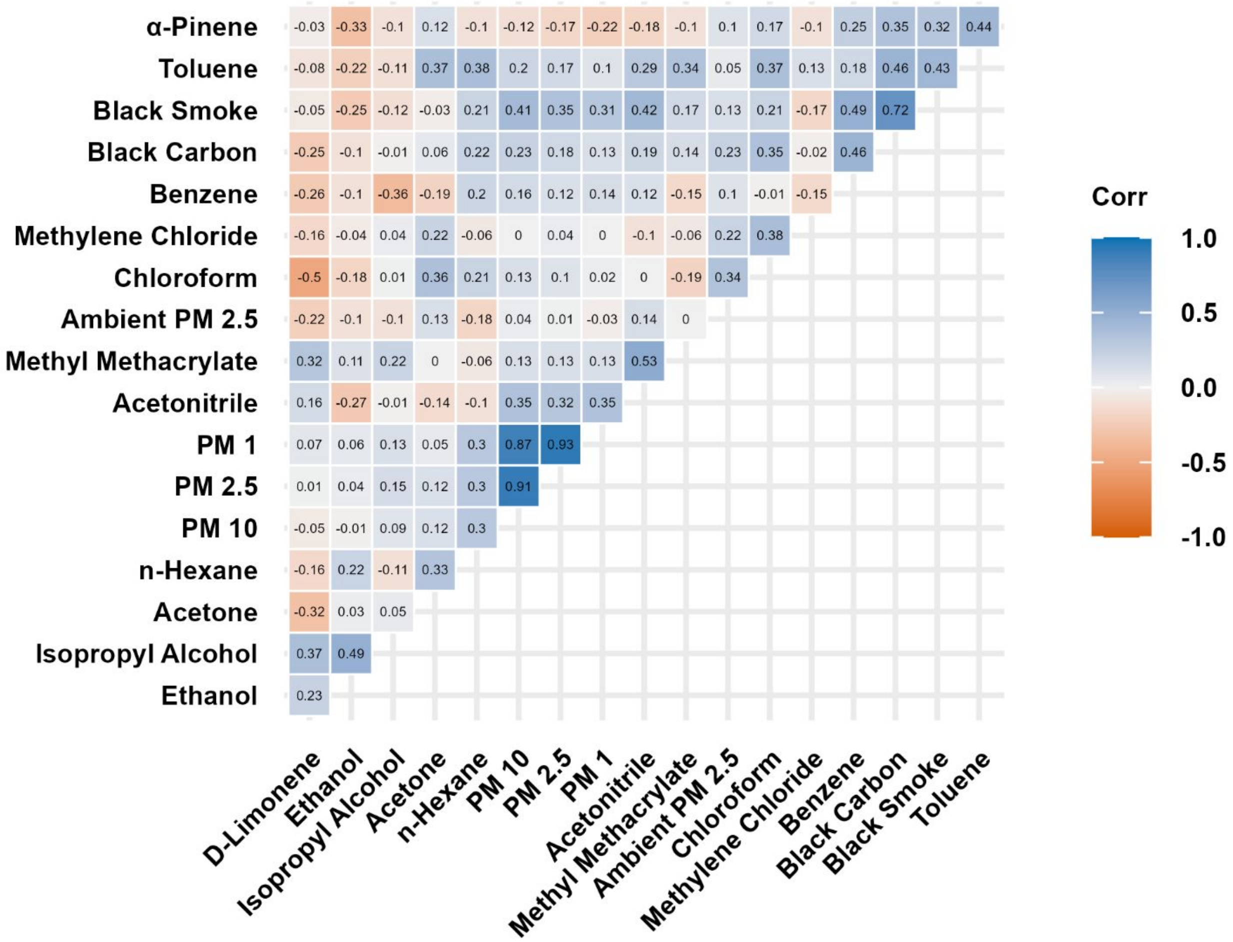

Figure S6. Kitchen Pollutant Correlation Matrix using a Kendall-Tau correlation plot for nonparametric data distribution ordered by hierarchical clustering. +1 suggests a positive correlation; 0 indicates no relationship; and -1 indicates an inverse relationship.
